# Supplementary material for: Beta-band dynamics during a naturalistic motor task: An OPM-MEG study
Source: Imaging Neurosci (Camb). 2026 Oct 22;4:IMAG.a.1405. doi: 10.1162/IMAG.a.1405 (PMC13697249; doi:10.1162/IMAG.a.1405)
Supplement: Supplementary file 1 [file IMAG.a.1405_supp.pdf]

Beta-band dynamics during a naturalistic motor task: an OPM-MEG study

Joseph Gibson[1], Jessikah Fildes[1], Alan Kirby[1], Niall Holmes[1,2], Lukas Rier[1,2], Karen J. Mullinger[1,3], Matias Ison[4], Peter G. Morris[1], Elena Boto[2,1], Ryan M. Hill[1,2] and Matthew J. Brookes[1,2]*

[1]Sir Peter Mansfield Imaging Centre, School of Physics and Astronomy, University of Nottingham, University Park, Nottingham, UK.

[2]Cerca Magnetics Limited, Units 7-8 Castlebridge Office Village, Kirtley Drive, Nottingham, UK

[3]Centre for Human Brain Health, School of Psychology, University of Birmingham, Edgbaston, Birmingham, UK

[4]School of Psychology, University of Nottingham, University Park, Nottingham, UK.

* Indicates correspondence to:

Prof. Matthew Brookes,
Sir Peter Mansfield Imaging Centre,
School of Physics and Astronomy,
University of Nottingham,
Nottingham
NG7 2RD
United Kingdom
E-mail: matthew.brookes@nottingham.ac.uk

Pages: 30
Words: 13,510
Figures: 7
Running title: Beta-band dynamics during a naturalistic motor task: an OPM-MEG study

KEYWORDS: Magnetoencephalography (MEG); Beta Oscillations; Motor Learning; Optically Pumped Magnetometers (OPMs); Violin; Functional Connectivity

SUPPLEMENTARY INFORMATION

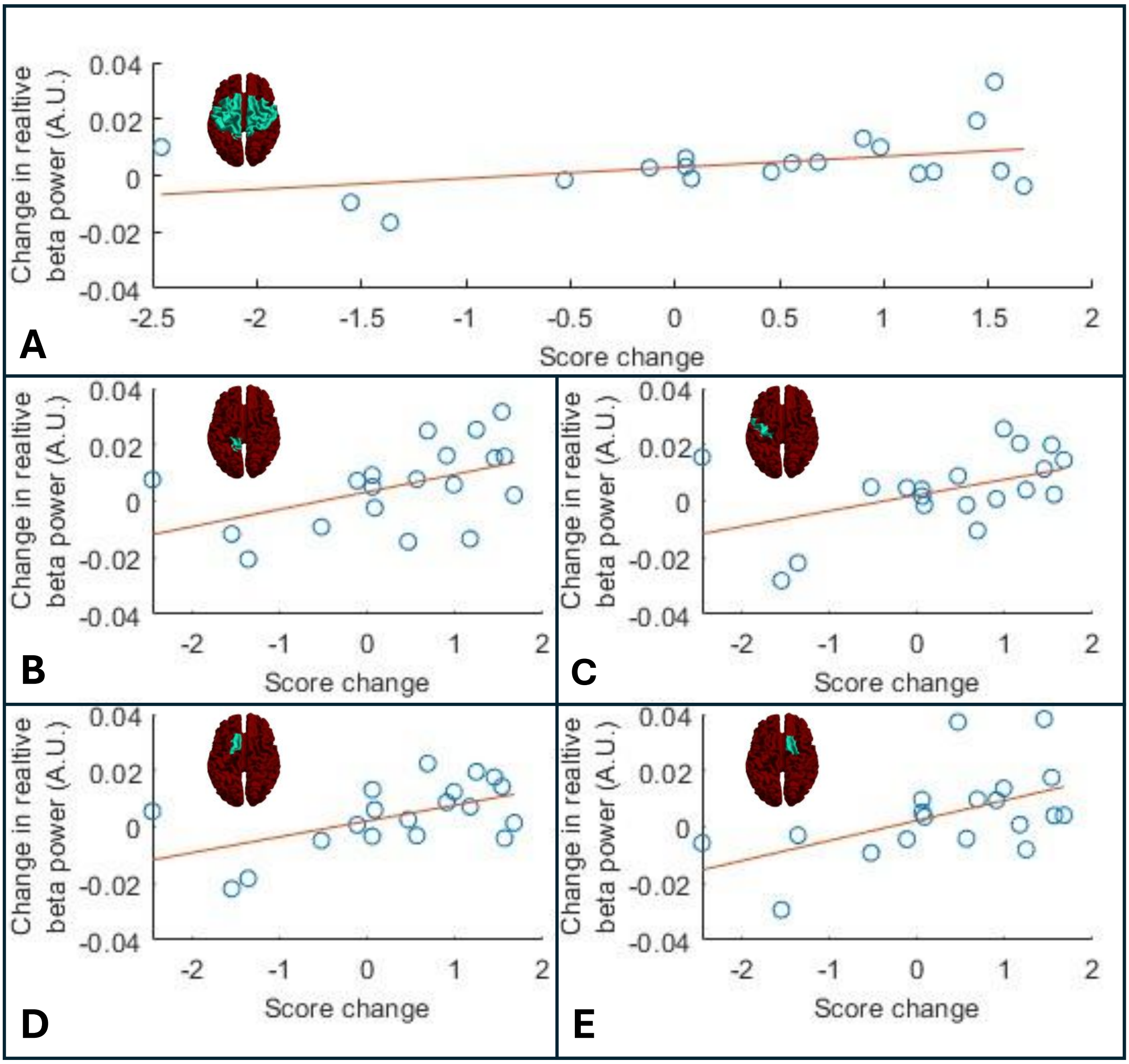

Figure S1: Correlations between the change in relative beta power during the playing window between sessions, and the change in trial averaged self-assessment score. *A) Beta power in the whole motor network (r = 0.3; p = 0.071). B) left dorsolateral motor cortex (r = 0.47; p = 0.04). C) left dorsomedial somatosensory cortex (r = 0.34; p = 0.034); D) Left dorsomedial premotor area (r = 0.55; p = 0.014). E) Right dorsomedial premotor area (r = 0.52; p = 0.02).*
